# Mandibular incisor alveolar housing and incisor-alveolar axis relationship in untreated skeletal Class III malocclusion: A cross-sectional CBCT study

**DOI:** 10.1371/journal.pone.0358261

**Published:** 2026-09-15

**Authors:** Thanh Huyen Nguyen, Huong Ly Nguyen, Thi Dung Dao, Thi Thu Trang Vu

**Affiliations:** 1 Department of Orthodontics, National Hospital of Odonto-Stomatology Hanoi, Hanoi, Vietnam; 2 Faculty of Dentistry, VNU University of Medicine and Pharmacy, Hanoi, Vietnam; 3 Department of Orthodontics, School of Dentistry, Hanoi Medical University, Hanoi, Vietnam; UCLA School of Dentistry, UNITED STATES OF AMERICA

## Abstract

Patients with skeletal Class III malocclusion may have limited alveolar support around the mandibular incisors, but conventional cephalometric measures do not directly describe the root-alveolus relationship. This retrospective cross-sectional study quantified labial and lingual alveolar bone thickness and examined whether signed IA-PAMD indicated apical root position within the symphysis. Pretreatment cone-beam computed tomography and lateral cephalometric records from 80 untreated patients (320 incisors) with signed ANB < 0° were analyzed. Bone thickness was measured at three root levels. Correlations and cluster-robust multivariable models adjusted for tooth, age, sex, signed ANB, GoGn-SN, and incisor mandibular plane angle. Mean labial thickness was 0.36, 0.49, and 2.40 mm at the cervical, middle, and apical levels; corresponding lingual values were 0.59, 1.39, and 4.01 mm. Labial bone was thinner than lingual bone (all P < 0.001), and labial thickness was below 0.5 mm in 80.3% of cervical and 63.4% of middle-root observations. Signed IA-PAMD correlated positively with labial apical thickness, negatively with lingual apical thickness, and not with total apical width. In adjusted models, each 1° increase in signed IA-PAMD was associated with 0.084 mm greater labial apical thickness (95% CI, 0.053–0.115), 0.092 mm lower lingual apical thickness (95% CI, −0.141 to −0.043), and no difference in total apical width (−0.008 mm; 95% CI, −0.082 to 0.065). The mirror-image labial and lingual coefficients, together with the near-zero total-width coefficient, support interpretation of signed IA-PAMD as a measure of root-apex eccentricity within the symphysis rather than aggregate apical bone support. It should not be interpreted as a treatment target or safety threshold, and the absence of a Class I comparator limits conclusions specific to skeletal Class III morphology.

## Introduction

Skeletal Class III malocclusion combines sagittal jaw disharmony with dentoalveolar compensation. Mandibular incisors commonly incline lingually, reducing the clinical severity of anterior crossbite but potentially placing their roots close to the cortical boundaries of the symphysis. Lower-incisor position is therefore relevant to camouflage treatment and presurgical decompensation, in which further tooth movement may alter periodontal support [1,2].

The incisor mandibular plane angle (IMPA) remains a convenient two-dimensional measure, but its reference is the mandibular plane rather than the local alveolar housing. The same IMPA can occur in symphyses with different width, inclination, and cortical distribution. Cone-beam computed tomography (CBCT) can directly depict the labial and lingual alveolar boundaries when three-dimensional imaging is already clinically indicated [3].

Previous CBCT studies have consistently reported reduced mandibular anterior alveolar dimensions or increased dehiscence and fenestration in skeletal Class III malocclusion, particularly in hyperdivergent subjects and during presurgical decompensation [4–17]. These studies differ in sampled teeth, skeletal subgroups, root levels, and defect definitions. Recent work has also emphasized that mean thickness alone may obscure the large proportion of sites at which the cortical plate is minimally detectable [10,11,16,17]. Thus, the present study is primarily confirmatory with respect to thin labial bone, but extends the evidence by examining all four mandibular incisors at three root levels in an untreated cohort and by reporting both patient-level asymmetry and tooth-level frequency distributions.

The angle between the lower-incisor long axis and the principal axis of the mandibular dentoalveolar bone (IA-PAMD) has been proposed as a local alternative or complement to IMPA [18]. Because IA-PAMD is referenced to the symphyseal alveolus rather than to the mandibular plane, it may capture information about root-alveolus alignment that IMPA does not. However, an unsigned angle can merge clinically opposite configurations, whereas a signed angle can distinguish whether the incisal portion of the tooth axis lies labial or lingual to the local symphyseal axis. A cross-sectional association should also not be interpreted as a biological limit for orthodontic movement.

This study aimed to quantify labial and lingual alveolar bone thickness around the four mandibular incisors in untreated skeletal Class III malocclusion, describe the frequency of measurements below 0.5 and 1.0 mm, and evaluate whether signed IA-PAMD was associated with the distribution of apical bone around the root apex beyond IMPA and skeletal covariates. The descriptive expectation that labial bone would be thinner than lingual bone preceded the current peer-review revision. The signed-angle three-outcome comparison was added during peer review to distinguish root-apex eccentricity from greater total apical support.

## Materials and methods

### Study design, setting, and ethics

This retrospective cross-sectional imaging study was reported according to the Strengthening the Reporting of Observational Studies in Epidemiology (STROBE) statement. Pretreatment wide-field CBCT and lateral cephalometric records acquired between June 2024 and June 2025 were identified among patients attending the Department of Orthodontics, Hanoi National Hospital of Odonto-Stomatology, Hanoi, Vietnam. The records were accessed for research purposes from June 1, 2025, to November 30, 2025. Only images acquired for clinical diagnosis or treatment planning were used; no additional imaging was performed for research purposes.

The authors had access to information that could identify individual patients during record screening and data extraction. Direct identifiers were subsequently removed and replaced with study codes before statistical analysis, and no identifying information was included in the analytical dataset. The study was approved by the Institutional Review Board for Ethics in Biomedical Research, VNU University of Medicine and Pharmacy, Hanoi, Vietnam (study code 1820; May 16, 2025). The Institutional Review Board waived the requirement for individual informed consent because the study involved retrospective analysis of existing diagnostic records that were de-identified before analysis.

### Participants

**Fig 1:**
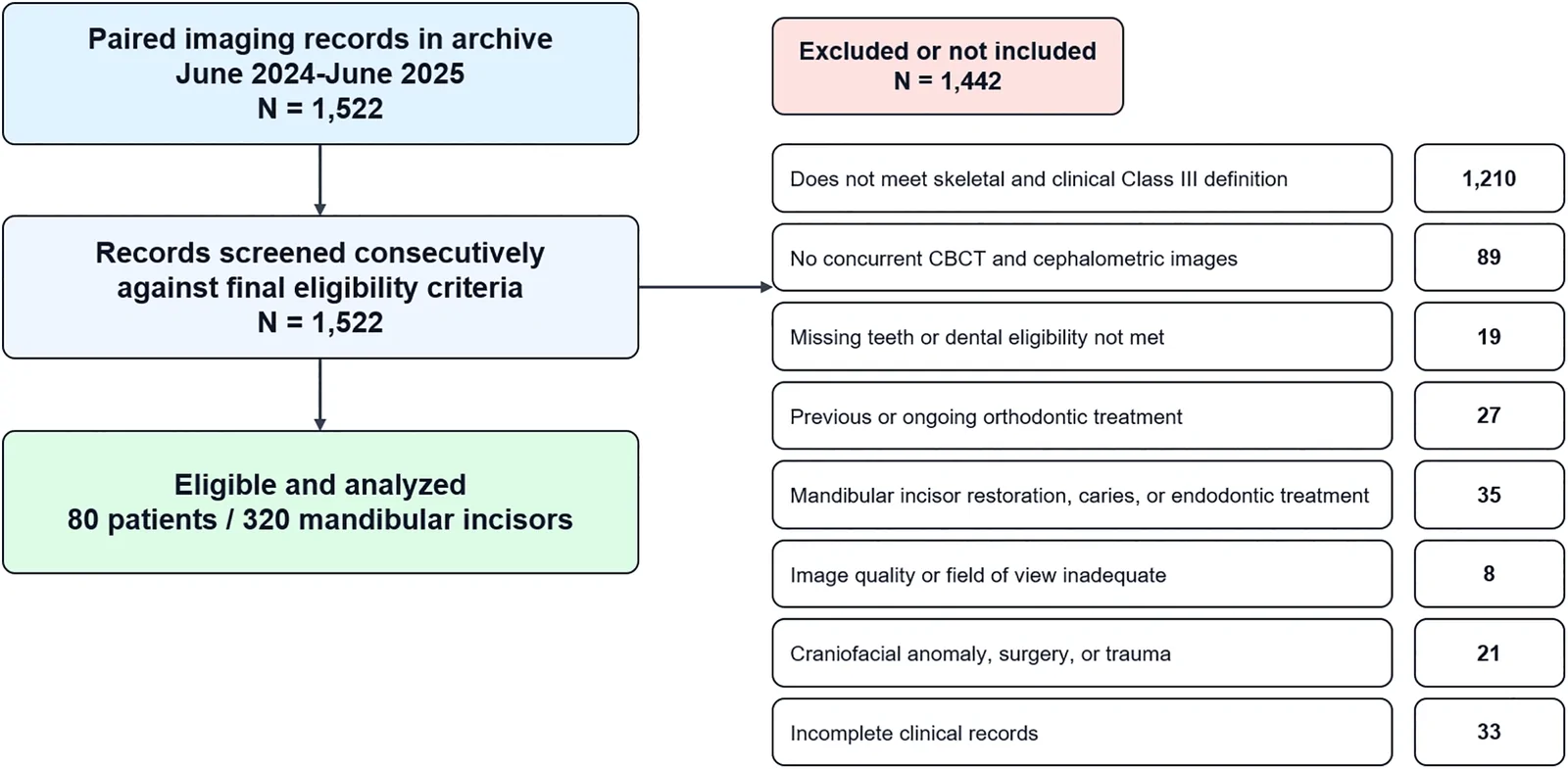
Participant screening flow. During the first-round revision, all 1,522 paired imaging records were re-screened consecutively using the revised final skeletal and clinical Class III definition. Each excluded record was assigned one primary final reason. Failure of the final Class III definition required consideration of signed ANB < 0° together with the specified clinical Class III occlusal criteria. The final analysis included 80 patients and 320 mandibular incisors.

The imaging archive contained 1,522 paired records acquired between June 2024 and June 2025. During the first-round revision, all 1,522 records were re-screened from the first record in the chronological archive using the revised final Class III definition, and screening continued until all records in the period had been reviewed. Of these, 1,442 records were excluded or not included for the primary reasons summarized in Fig 1, leaving 80 eligible patients for analysis. The current sample therefore reflects complete revision-stage re-screening and was not obtained by stopping once a target number had been reached.

As a post hoc study-size justification for the secondary association analysis, a previously reported correlation of r = 0.463 between lower-incisor inclination and buccal apical alveolar bone thickness [19] was used. A minimum of 45 independent participants would provide 90% power at a two-sided alpha of 0.05 for a correlation of this magnitude. This calculation was added during peer review, was not used to stop enrolment, and did not determine which records were included; all eligible patients in the study period were retained.

Eligibility criteria were: pretreatment wide-field CBCT and lateral cephalometric images acquired at the same diagnostic stage; 28 permanent teeth excluding third molars, with the teeth required to be present on the images rather than necessarily fully erupted; adequate image quality and a field of view of 13 x 13 cm; complete apical closure of all four mandibular incisors; and skeletal Class III diagnosis defined by signed ANB < 0° together with a clinical Class III canine or molar relationship and anterior edge-to-edge bite or anterior crossbite. The source master’s-thesis cohort had originally used sex-specific Wits appraisal criteria. For the first-round revision, however, Wits appraisal was removed from the case definition and the revised skeletal and clinical criteria just described were applied uniformly during re-screening of all 1,522 records. Wits values were not retained in the revised analytic dataset and were therefore not used in the revision-stage re-screening or the current analysis. Exclusion criteria were previous or ongoing orthodontic treatment; restorations, proximal caries, or endodontic treatment affecting mandibular incisors; abnormal incisor morphology or position that precluded standardized measurement; congenital craniofacial anomalies; previous orthognathic surgery; and maxillofacial trauma.

### CBCT acquisition and image orientation

CBCT examinations were obtained in the hospital imaging department with a Planmeca ProMax 3D Max unit (Planmeca, Helsinki, Finland). All scans used a single acquisition protocol performed by trained technicians: 96 kV, 5.6 mA, 12.08-second scan time, 13 x 13 cm field of view, and 0.20-mm voxel size. Digital Imaging and Communications in Medicine data were imported into InVivo Dental 6/6.5 (Anatomage, San Jose, CA, USA). Images were oriented in a standardized coordinate system using the midsagittal plane and a horizontal plane based on bilateral orbital landmarks. For each incisor, the sagittal section was adjusted to pass through the center of the crown and root and was checked in the axial and coronal views before measurement.

### Alveolar bone and axis measurements

The mandibular incisors were coded according to the FDI system (32, 31, 41, and 42). The long axis of each tooth was defined from the incisal midpoint to the root apex. A reference segment extending from the intersection of the tooth axis with the cementoenamel-junction level to the root apex was divided into three equal parts. The cervical and middle measurement levels were located at the first and second division points from the cementoenamel-junction level, respectively. At the cervical and middle levels, labial and lingual alveolar bone thicknesses were measured from the root surface to the outer cortical boundary along lines perpendicular to the tooth axis. Apical thickness was measured from the root apex to the labial or lingual cortical boundary along the corresponding apical line, which was perpendicular to the tooth axis and parallel to the cervical and middle measurement lines. A value of 0 mm indicated that a cortical plate could not be resolved at the measurement point.

**Fig 2:**
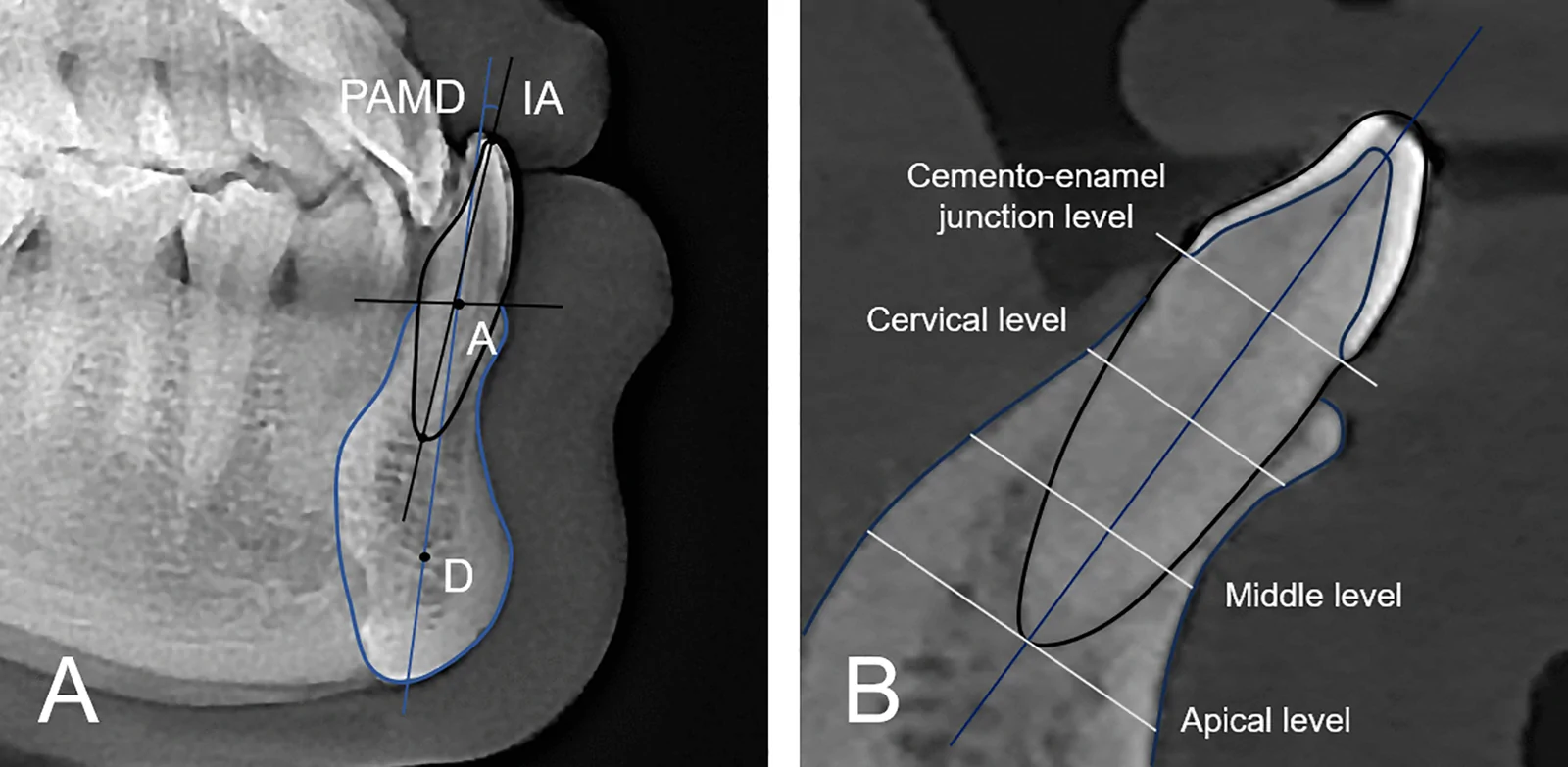
Signed IA-PAMD and alveolar bone thickness measurements. (A) IA is the mandibular incisor long axis; point A is the midpoint of the line joining the labial and lingual alveolar crests; point D is the visual geometric center of the mandibular symphyseal bony outline; and PAMD is the line joining A and D. Signed IA-PAMD is positive when the incisal portion of the tooth axis lies labial to PAMD and negative when it lies lingual to PAMD. (B) The reference segment from the tooth-axis intersection at the cementoenamel-junction level to the root apex is divided into three equal parts. The cervical and middle levels are the first and second division points, and the apical line passes through the root apex. All three bone-thickness measurement lines are perpendicular to the tooth axis and parallel to one another.

Signed IA-PAMD was constructed on the same standardized sagittal section (Fig 2). IA was the incisor long axis. A line was drawn between the labial and lingual alveolar crests adjacent to the measured incisor, and point A was defined as the midpoint of this alveolar-crest line. Point D was operationally defined as the visual geometric center of the mandibular symphyseal bony outline on that sagittal section. PAMD was the line joining point A to point D. Signed IA-PAMD was recorded in degrees. Positive values indicated that the incisal portion of the tooth axis lay labial to PAMD, corresponding to relative proclination and a root apex displaced toward the lingual cortex. Negative values indicated that the incisal portion of the tooth axis lay lingual to PAMD, corresponding to relative retroclination and a root apex displaced toward the labial cortex. The PAMD construction followed the published local-axis concept [18], with the present protocol adding the signed convention and explicit operational definitions of points A and D. The term principal axis is used here in this operational sense rather than as a mathematically derived principal component.

Conventional cephalometric variables were SNA, SNB, signed ANB calculated as SNA minus SNB, GoGn-SN, and IMPA. All measurements were performed without access to treatment outcomes because the images were pretreatment records.

### Measurement reliability

Two calibrated examiners independently repeated the measurement protocol in the same 10 randomly selected CBCT scans (40 incisors) and were blinded to each other’s results. For intra-rater reliability, Examiner 1 repeated measurements on those same 40 incisors after a 2-week interval while blinded to the first measurements. The same image-orientation rules, landmark definitions, calibration procedure, and analysis unit were used for the inter- and intra-rater comparisons. Inter-rater reliability was assessed using a two-way random-effects, absolute-agreement, single-measure intraclass correlation coefficient (ICC). Intra-rater reliability was assessed using a two-way mixed-effects, absolute-agreement, single-measure ICC. The 95% confidence intervals for both sets of ICCs were obtained with the same tooth-level bootstrap procedure. Absolute disagreement was summarized as mean absolute difference and Bland-Altman limits of agreement. Reliability analyses were reported separately for labial and lingual thickness at the cervical, middle, and apical levels and for signed IA-PAMD. The small numerical differences between inter- and intra-rater estimates were not interpreted as a ranking of reproducibility.

### Statistical analysis

Continuous data are presented as mean and standard deviation or as median and interquartile range, as appropriate, and categorical data as frequency and percentage. Tooth-level measurements were summarized by tooth and root level. Because four incisors were nested within each participant, labial and lingual thicknesses were first averaged across the four teeth for each participant; these patient-level averages were compared using two-sided Wilcoxon signed-rank tests. Medians and interquartile ranges were reported together with Wilcoxon test statistics and effect sizes, calculated as the absolute value of the standardized Wilcoxon z statistic divided by the square root of the number of paired observations.

Tooth-level thicknesses were additionally categorized as <0.5 mm and <1.0 mm to describe the frequency of minimal radiographically detectable bone and thin bone, respectively. These cutoffs were used as descriptive imaging categories and were not treated as validated biological safety thresholds.

Tooth-specific Spearman rank correlations assessed the associations between signed IA-PAMD and three apical outcomes: labial apical thickness, lingual apical thickness, and total apical width (labial plus lingual). For each outcome, the four tooth-specific correlation P values were adjusted with the Holm method. Multivariable analyses used each apical outcome as the dependent variable and signed IA-PAMD as the main independent variable. Models adjusted for tooth, age, sex, signed ANB, GoGn-SN, and IMPA. Ordinary least-squares coefficients were estimated with participant-clustered robust standard errors.

To examine whether signed IA-PAMD improved explanatory fit within this dataset beyond the conventional covariates, model R-squared was compared before and after inclusion of signed IA-PAMD, and partial R-squared was calculated. The adult-only sensitivity analysis had been specified before the current peer-review revision. The signed-angle three-outcome correlations and models, the stricter signed ANB<=−2° sensitivity subset, and the correlation-based study-size justification were added during peer review to address interpretation and case-definition questions. These revision analyses were therefore treated as secondary or sensitivity analyses rather than as prospectively specified confirmatory tests.

All tests were two-sided with alpha = 0.05. Analyses were performed in Python 3.11 using SciPy and statsmodels. No values were imputed. Data were double-entered. The de-identified analysis dataset used for the revised analyses is provided as S1 File.

## Results

### Participant characteristics

The final sample included 80 untreated patients and 320 mandibular incisors. The sample comprised 38 males (47.5%) and 42 females (52.5%), with a mean age of 18.31 years (standard deviation 4.77; range 11−33). Thirty-nine participants (48.8%) were adults aged at least 18 years. The mean signed ANB was −3.83 degrees; values ranged from −12.31 to −0.52 degrees, with a median of −3.69 degrees and interquartile range of −5.15 to −2.19 degrees. Sixty-three participants had signed ANB<=−2° (Table 1).

**Table 1:** Participant characteristics (n = 80).

Characteristic	Value
Age, years	18.31 ± 4.77 (11-33)
Age 11–14 years	17 (21.2%)
Age 15–17 years	24 (30.0%)
Age >=18 years	39 (48.8%)
Male sex	38 (47.5%)
Female sex	42 (52.5%)
SNA, degrees	83.22 ± 3.87
SNB, degrees	87.05 ± 4.09
Signed ANB, degrees	−3.83 ± 2.18
GoGn-SN, degrees	30.95 ± 6.21
IMPA, degrees	91.72 ± 7.20

Note. Values are mean ± standard deviation (range) or n (%). IMPA, incisor mandibular plane angle.

### Alveolar bone thickness

**Fig 3:**
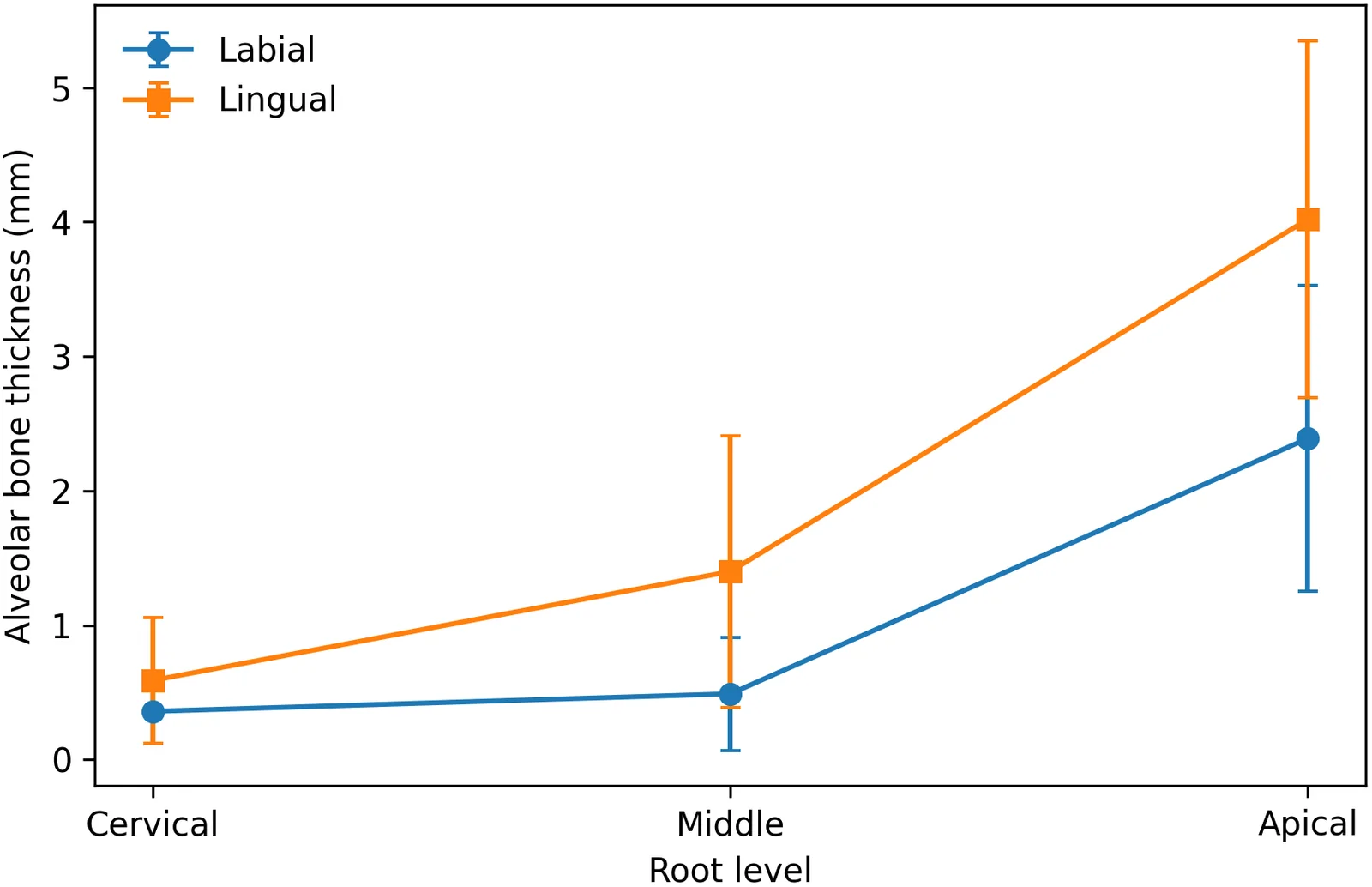
Mean labial and lingual alveolar bone thickness by root level. Error bars indicate standard deviations.

Across the four incisors, the labial plate was thinnest at the cervical and middle root levels. Tooth-specific mean labial cervical thickness ranged from 0.34 to 0.40 mm, whereas labial middle thickness ranged from 0.46 to 0.52 mm. At the apex, mean labial thickness ranged from 2.18 to 2.59 mm. The lingual plate increased more markedly from the cervical level to the apex, where tooth-specific means ranged from 3.92 to 4.14 mm (Table 2 and Fig 3).

**Table 2:** Tooth-specific alveolar bone thickness.

Tooth	Surface	Cervical, mm	Middle, mm	Apical, mm
32	Labial	0.40 ± 0.26	0.50 ± 0.41	2.59 ± 1.14
32	Lingual	0.73 ± 0.58	1.49 ± 1.01	3.92 ± 1.27
31	Labial	0.36 ± 0.21	0.46 ± 0.38	2.18 ± 1.11
31	Lingual	0.51 ± 0.36	1.38 ± 1.15	4.14 ± 1.37
41	Labial	0.35 ± 0.25	0.48 ± 0.42	2.30 ± 1.20
41	Lingual	0.53 ± 0.44	1.26 ± 0.90	4.04 ± 1.35
42	Labial	0.34 ± 0.22	0.52 ± 0.49	2.53 ± 1.11
42	Lingual	0.59 ± 0.47	1.43 ± 0.98	3.94 ± 1.38

Note. Values are mean ± standard deviation. Tooth numbers follow FDI notation; n = 80 measurements for each tooth.

When the four incisors were averaged within each participant, labial bone was thinner than lingual bone at all root levels. Median lingual-labial differences increased from 0.12 mm cervically to 0.64 mm at the middle level and 1.44 mm apically (all P < 0.001; Table 3). This result describes asymmetry of the observed housing but does not by itself establish the direction or amount of permissible tooth movement.

**Table 3:** Patient-averaged labial and lingual alveolar bone thickness.

Root level	Labial, mm	Lingual, mm	Lingual-labial difference, mm	Wilcoxon W	P value	Effect size r
Cervical	0.34 (0.26-0.44)	0.48 (0.32-0.71)	0.12 (−0.05-0.41)	770.0	<0.001	0.456
Middle	0.42 (0.25-0.58)	1.06 (0.71-1.84)	0.64 (0.37-1.29)	83.0	<0.001	0.824
Apical	2.27 (1.65-2.94)	3.72 (3.08-4.81)	1.44 (0.76-2.42)	146.0	<0.001	0.790

Note. Values were averaged across the four incisors within each participant before comparison. Values are median (interquartile range) unless otherwise stated. P values are from two-sided Wilcoxon signed-rank tests.

### Frequency of thin-bone measurements

At the tooth level, labial cervical thickness was below 0.5 mm in 257 of 320 observations (80.3%) and below 1.0 mm in 312 observations (97.5%). At the labial middle level, the corresponding frequencies were 63.4% and 92.2%. Only 6.6% of labial apical measurements were below 1.0 mm. Measurements of 0 mm were recorded in 25 labial cervical, 22 labial middle, and one labial apical observations; corresponding lingual counts were 25 cervical, five middle, and none apically (Table 4).

**Table 4:** Tooth-level frequency of alveolar bone measurements below 0.5 and 1.0 mm.

Surface	Root level	n	0 mm, n (%)	<0.5 mm, n (%)	<1.0 mm, n (%)	Mean ± SD, mm
Labial	Cervical	320	25 (7.8%)	257 (80.3%)	312 (97.5%)	0.36 ± 0.24
Labial	Middle	320	22 (6.9%)	203 (63.4%)	295 (92.2%)	0.49 ± 0.42
Labial	Apical	320	1 (0.3%)	3 (0.9%)	21 (6.6%)	2.40 ± 1.15
Lingual	Cervical	320	25 (7.8%)	169 (52.8%)	270 (84.4%)	0.59 ± 0.47
Lingual	Middle	320	5 (1.6%)	52 (16.2%)	147 (45.9%)	1.39 ± 1.01
Lingual	Apical	320	0 (0.0%)	0 (0.0%)	0 (0.0%)	4.01 ± 1.34

Note. The thresholds are descriptive CBCT categories, not validated limits for orthodontic movement. A value of 0 mm indicates that a cortical plate could not be resolved at the measurement point.

### Association between signed IA-PAMD and apical bone distribution

Mean signed IA-PAMD ranged from 5.11 degrees at tooth 31 to 7.12 degrees at tooth 32. Negative signed IA-PAMD values occurred in 45 of 320 observations (14.1%), with an overall observed range of −15.5° to 22.5°. By tooth, negative values occurred in 8 of 80 observations (10.0%) for tooth 32, 14 of 80 (17.5%) for tooth 31, 11 of 80 (13.8%) for tooth 41, and 12 of 80 (15.0%) for tooth 42; corresponding observed ranges were −10.0° to 22.5°, −15.5° to 20.9°, −13.7° to 20.3°, and −6.8° to 21.5°, respectively. Signed IA-PAMD was positively correlated with labial apical thickness for all four incisors, with rho values from 0.381 to 0.617. The same angle was negatively correlated with lingual apical thickness and showed no significant association with total apical width (Table 5).

**Table 5:** Tooth-specific signed IA-PAMD and correlations with apical bone dimensions.

Tooth	Signed IA-PAMD, degrees	Labial apical rho (Holm P)	Lingual apical rho (Holm P)	Total apical width rho (Holm P)
32	7.12 ± 6.25	0.445 (<0.001)	−0.358 (0.004)	0.006 (1.000)
31	5.11 ± 6.05	0.617 (<0.001)	−0.321 (0.011)	0.121 (1.000)
41	5.73 ± 6.01	0.448 (<0.001)	−0.315 (0.011)	0.046 (1.000)
42	6.65 ± 6.10	0.381 (<0.001)	−0.308 (0.011)	−0.007 (1.000)

Note. Values are mean ± standard deviation unless otherwise stated. P values in parentheses are Holm-adjusted within each outcome. IA-PAMD, incisor axis-principal axis of the mandibular dentoalveolar bone angle.

Adjusted models confirmed this mirror-image pattern. Each 1-degree higher signed IA-PAMD was associated with 0.084 mm greater labial apical thickness (95% CI, 0.053 to 0.115; P < 0.001) and 0.092 mm lower lingual apical thickness (95% CI, −0.141 to −0.043; P < 0.001). In contrast, the coefficient for total apical width was close to zero and not statistically significant (coefficient, −0.008 mm; 95% CI, −0.082 to 0.065; P = 0.824). Adding signed IA-PAMD increased R² by 0.178 for labial apical thickness and by 0.157 for lingual apical thickness, but by only 0.001 for total apical width (Table 6). Expressed per one standard deviation of signed IA-PAMD, the adjusted coefficients corresponded to 0.512 mm greater labial apical thickness, 0.563 mm lower lingual apical thickness, and 0.051 mm lower total apical width.

**Table 6:** Cluster-robust multivariable models for apical alveolar dimensions.

Outcome	Signed IA-PAMD coefficient, mm/degree	95% CI	P value	Model R²	Delta R²	Partial R²
Labial apical thickness	0.084	0.053 to 0.115	<0.001	0.335	0.178	0.211
Lingual apical thickness	−0.092	−0.141 to −0.043	<0.001	0.313	0.157	0.186
Total apical width	−0.008	−0.082 to 0.065	0.824	0.226	0.001	0.001

Note. Each model adjusted for tooth, age, sex, signed ANB, GoGn-SN, and IMPA; standard errors were clustered by participant. Full-sample n = 320 teeth from 80 participants. Delta R² is the increase after adding signed IA-PAMD to the covariate-only model. CI, confidence interval; IMPA, incisor mandibular plane angle.

The pattern persisted in sensitivity analyses. In adults only (39 participants, 156 incisors), signed IA-PAMD coefficients were 0.079 mm/degree for labial apical thickness (95% CI, 0.044 to 0.114; P < 0.001), −0.082 mm/degree for lingual apical thickness (95% CI, −0.137 to −0.027; P = 0.003), and −0.003 mm/degree for total apical width (95% CI, −0.084 to 0.078; P = 0.938). In the signed ANB<=−2° subset (63 participants, 252 incisors), the corresponding coefficients were 0.086 mm/degree (95% CI, 0.045 to 0.127; P < 0.001), −0.075 mm/degree (95% CI, −0.136 to −0.015; P = 0.015), and 0.011 mm/degree (95% CI, −0.080 to 0.102; P = 0.812).

### Measurement reliability

Relative reliability was high, although absolute disagreement was non-negligible for some measurements. Intra-rater ICCs ranged from 0.919 to 0.935 and inter-rater ICCs from 0.916 to 0.929. Mean absolute differences for thickness measurements ranged from 0.09 to 0.54 mm intra-rater and from 0.09 to 0.56 mm inter-rater; for signed IA-PAMD, mean absolute differences were 2.22° intra-rater and 2.56° inter-rater (Table 7).

**Table 7:** Inter-rater and intra-rater reliability.

Measurement	Intra-rater ICC (95% CI)	Intra-rater MAD	Intra-rater LoA	Inter-rater ICC (95% CI)	Inter-rater MAD	Inter-rater LoA
Labial cervical	0.919 (0.831-0.960)	0.09 mm	−0.23 to 0.22 mm	0.916 (0.782-0.960)	0.09 mm	−0.24 to 0.22 mm
Lingual cervical	0.924 (0.850-0.957)	0.19 mm	−0.46 to 0.48 mm	0.921 (0.839-0.957)	0.19 mm	−0.46 to 0.51 mm
Labial middle	0.926 (0.805-0.957)	0.27 mm	−0.56 to 0.70 mm	0.923 (0.810-0.959)	0.28 mm	−0.58 to 0.73 mm
Lingual middle	0.929 (0.883-0.954)	0.38 mm	−0.92 to 0.92 mm	0.926 (0.875-0.954)	0.42 mm	−0.98 to 1.04 mm
Labial apical	0.932 (0.857-0.962)	0.52 mm	−1.30 to 1.22 mm	0.919 (0.832-0.952)	0.55 mm	−1.35 to 1.23 mm
Lingual apical	0.933 (0.885-0.957)	0.54 mm	−1.30 to 1.26 mm	0.927 (0.869-0.955)	0.56 mm	−1.34 to 1.29 mm
Signed IA-PAMD	0.935 (0.896-0.957)	2.22 °	−5.37 to 5.10 °	0.929 (0.882-0.952)	2.56 °	−6.16 to 5.89 °

Note. Inter-rater ICC used a two-way random-effects, absolute-agreement, single-measure model. Intra-rater ICC used a two-way mixed-effects, absolute-agreement, single-measure model. Confidence intervals are bootstrap percentile intervals. MAD, mean absolute difference; LoA, Bland-Altman limits of agreement.

## Discussion

This study found a consistent labial-lingual asymmetry around untreated mandibular incisors in skeletal Class III malocclusion. The labial plate was frequently below 0.5 mm at cervical and middle root levels, whereas the lingual plate was thicker, particularly at the apex. The revised signed-angle analysis changed the interpretation of IA-PAMD. A larger signed IA-PAMD was associated with greater labial apical thickness and lower lingual apical thickness, while total apical width was essentially unchanged. Signed IA-PAMD is therefore better interpreted as an indicator of root-apex eccentricity within the symphysis than as a correlate of aggregate apical bone support.

The thin labial plate and greater lingual support are consistent with prior CBCT studies of skeletal Class III samples [4–17]. Kim et al. and Kook et al. described reduced incisor alveolar support in surgical Class III patients [4,5]. Studies assessing sagittal and vertical subtypes subsequently showed that hyperdivergence is associated with narrower mandibular anterior housing and a greater prevalence of dehiscence or fenestration [6–9,16]. More recent studies have confirmed frequent anterior bone defects across malocclusion groups and in untreated Class III cohorts [10,11,17]. Accordingly, the descriptive morphology in the present study should not be presented as entirely novel. Its contribution lies in the tooth-specific assessment of all four mandibular incisors, patient-level quantification of labial-lingual asymmetry, complete frequency reporting at three root levels, and analysis of a clearly defined local axis measure.

The clinical interpretation of measurements near or below the CBCT voxel dimension requires caution. With a voxel size of 0.20 mm, a reported thickness below 0.5 mm represents only a small number of voxels and is susceptible to partial-volume effects, image noise, and threshold selection. We therefore interpreted <0.5 mm as minimally detectable radiographic bone rather than an exact histologic thickness. Similarly, the 1.0-mm category describes thin bone but should not be treated as a universal threshold for safe tooth movement. At the labial cervical level, the intra-rater and inter-rater Bland-Altman limits of agreement were −0.23 to 0.22 mm and −0.24 to 0.22 mm, respectively, both of the same order as the mean thickness (0.36 mm), illustrating that stable group summaries do not imply equivalent precision for a single very thin cortical measurement. The mean absolute differences for labial apical thickness were 0.52 mm intra-rater and 0.55 mm inter-rater. Individual treatment decisions should therefore combine direct image assessment with the periodontal phenotype and other clinical findings rather than rely on a single numeric thickness threshold.

Signed IA-PAMD relates the incisor axis to the local symphyseal axis rather than to the mandibular plane [18]. Its clinical value is not that it measures more bone in absolute terms, but that it summarizes where the root apex lies between the labial and lingual cortical plates on the standardized sagittal section. A larger positive signed IA-PAMD reflects displacement of the root apex toward the lingual cortex relative to the local axis, with greater labial and lower lingual apical thickness; a shift in the opposite direction produces the reverse pattern. The essentially unchanged total apical width indicates redistribution of the root position within the available buccolingual housing rather than creation of greater apical support. Accordingly, signed IA-PAMD is a descriptive local-axis indicator and should not be pursued as a target angular value during decompensation.

The age range was a legitimate source of heterogeneity. Growth and maturation can influence symphyseal morphology, and chronological age is an imperfect proxy for skeletal maturity. Rather than excluding adolescents from the primary description, we adjusted for age and repeated the complete models in adults only. The same labial-positive, lingual-negative, total-width-null pattern was retained. All mandibular incisors had complete apical closure on CBCT, including in participants aged 11–17 years.

Longitudinal studies have shown that presurgical decompensation may reduce alveolar thickness and increase bone defects, particularly in hyperdivergent Class III patients [20–24]. Those findings provide the clinical context for measuring the initial alveolar housing, but the present cross-sectional data do not predict bone response to orthodontic movement. The signed IA-PAMD association describes baseline root-apex position within the existing symphysis.

### Strengths and limitations

Strengths include a standardized single-center acquisition protocol, evaluation of all four mandibular incisors, explicit operational definition of signed IA-PAMD, reporting of both relative and absolute reliability metrics, analysis of clustered tooth-level data, multiplicity adjustment for tooth-specific correlations, complete data availability, and sensitivity analyses in adults and in a stricter ANB subset.

The principal limitations are the retrospective cross-sectional design, absence of a Class I control group, single-center sampling, and lack of longitudinal treatment or periodontal outcomes. The study cannot determine whether moving an incisor labially or lingually would preserve, increase, or reduce cortical bone. The 0.5-mm and 1.0-mm categories are descriptive CBCT categories rather than validated biological thresholds. Measurements close to the 0.20-mm voxel size should be interpreted cautiously because partial-volume effects, image noise, and threshold selection may affect visibility of very thin cortices.

Finally, signed IA-PAMD is influenced by symphyseal morphology because PAMD is anchored to the visual geometric center of the mandibular symphysis. Variations in symphyseal height, width, inclination, and chin prominence may alter PAMD orientation independently of local alveolar thickness. Point D is also an operator-defined visual landmark rather than an automatically derived geometric centroid; its reproducibility therefore depends on image orientation, calibration, and examiner experience. Although reliability was high under the present single-center protocol, multicenter use would require explicit landmark standardization, examiner training, and independent reproducibility testing. Accordingly, signed IA-PAMD should be used as a constructed local-axis measure of root-alveolus relationship, not as a standalone index of alveolar housing validity or treatment safety.

## Conclusions

In this cohort of untreated patients with skeletal Class III malocclusion, mandibular incisor alveolar housing was asymmetric: the labial plate was frequently minimally detectable at cervical and middle root levels, whereas the lingual plate was thicker, especially apically. Signed IA-PAMD described the labiolingual eccentricity of the incisor root apex within the symphysis. Larger signed IA-PAMD values were associated with greater labial and lower lingual apical thickness, but not with greater total apical width. Signed IA-PAMD should therefore be interpreted as a local axis relationship and not as a treatment target, a treatment-safety threshold, or a measure of aggregate apical bone support.

## Supporting information

S1 File De-identified tooth-level analysis dataset. (XLSX)

S2 File STROBE checklist. (DOCX)
