# Supplementary material for: Mandibular incisor alveolar housing and incisor-alveolar axis relationship in untreated skeletal Class III malocclusion: A cross-sectional CBCT study
Source: PLoS One. 2026 Sep 15;21(9):e0358261. doi: 10.1371/journal.pone.0358261 (PMC13577396; doi:10.1371/journal.pone.0358261)
Supplement: Supplementary file 1 [file pone.0358261.s002.docx]

**STROBE Statement - Checklist for Cross-Sectional Studies**

**Manuscript: Mandibular incisor alveolar housing and incisor-alveolar axis relationship in untreated skeletal Class III malocclusion: a cross-sectional CBCT study**

| **Section / topic** | **Item** | **Recommendation** | **Reported on page No.** |
| --- | --- | --- | --- |
| **Title and abstract** | **1a** | Indicate the study design with a commonly used term in the title or the abstract. | Title, p. 1; Abstract, p. 2 |
|  | **1b** | Provide in the abstract an informative and balanced summary of what was done and what was found. | Abstract, p. 2 |
| **Introduction - Background/rationale** | **2** | Explain the scientific background and rationale for the investigation being reported. | pp. 3-4 |
| **Introduction - Objectives** | **3** | State specific objectives, including any prespecified hypotheses. | p. 4 |
| **Methods - Study design** | **4** | Present key elements of the study design early in the paper. | p. 4 |
| **Methods - Setting** | **5** | Describe the setting, locations, and relevant dates, including periods of recruitment, exposure, follow-up, and data collection. | pp. 4-5 |
| **Methods - Participants** | **6a** | Give the eligibility criteria and the sources and methods of selection of participants. | pp. 5-6; Fig 1 |
|  | **6b** | For matched studies, give matching criteria and the number of exposed and unexposed participants. | Not applicable (unmatched cross-sectional study) |
| **Methods - Variables** | **7** | Clearly define outcomes, exposures, predictors, potential confounders, and effect modifiers. Give diagnostic criteria, when applicable. | pp. 5-10 |
| **Methods - Data sources/measurement** | **8** | For each variable of interest, give sources of data and details of assessment methods. Describe comparability of assessment methods if there is more than one group. | pp. 6-9; Fig 2 |
| **Methods - Bias** | **9** | Describe any efforts to address potential sources of bias. | pp. 4-10 and 18-19 |
| **Methods - Study size** | **10** | Explain how the study size was arrived at. | pp. 5-6 |
| **Methods - Quantitative variables** | **11** | Explain how quantitative variables were handled in the analyses. If applicable, describe which groupings were chosen and why. | pp. 9-10 |
| **Methods - Statistical methods** | **12a** | Describe all statistical methods, including those used to control for confounding. | pp. 9-11 |
|  | **12b** | Describe any methods used to examine subgroups and interactions. | pp. 10 and 15 (adult-only and stricter ANB sensitivity analyses) |
|  | **12c** | Explain how missing data were addressed. | p. 11 (no imputation; complete revised analysis dataset) |
|  | **12d** | For cross-sectional studies, describe analytical methods taking account of the sampling strategy. | pp. 5-6 and 10 (all eligible records retained; participant-clustered robust standard errors) |
|  | **12e** | Describe any sensitivity analyses. | pp. 10 and 15 |
| **Results - Participants** | **13a** | Report numbers of individuals at each stage of the study, for example potentially eligible, examined for eligibility, confirmed eligible, included, and analysed. | pp. 5-6 and 11; Fig 1 |
|  | **13b** | Give reasons for non-participation at each stage. | pp. 5-6; Fig 1 |
|  | **13c** | Consider use of a flow diagram. | p. 6; Fig 1 |
| **Results - Descriptive data** | **14a** | Give characteristics of study participants and information on exposures and potential confounders. | p. 11; Table 1 |
|  | **14b** | Indicate the number of participants with missing data for each variable of interest. | p. 11 (no values imputed; complete-case analysis dataset) |
|  | **14c** | Summarise follow-up time. | Not applicable (cross-sectional study) |
| **Results - Outcome data** | **15** | Report numbers of outcome events or summary measures. | pp. 11-15; Tables 2-5; Fig 3 |
| **Results - Main results** | **16a** | Give unadjusted estimates and, if applicable, confounder-adjusted estimates and their precision. Make clear which confounders were adjusted for and why they were included. | pp. 14-15; Tables 5-6 |
|  | **16b** | Report category boundaries when continuous variables were categorised. | pp. 10 and 13; Table 4 (<0.5 mm and <1.0 mm categories) |
|  | **16c** | If relevant, consider translating estimates of relative risk into absolute risk for a meaningful time period. | Not applicable (no risk or incidence estimates) |
| **Results - Other analyses** | **17** | Report other analyses done, for example subgroup analyses, sensitivity analyses, and measurement-reliability analyses. | pp. 15-16; Table 7 |
| **Discussion - Key results** | **18** | Summarise key results with reference to study objectives. | pp. 16-19 |
| **Discussion - Limitations** | **19** | Discuss limitations, taking into account sources of potential bias or imprecision. Discuss both direction and magnitude of potential bias. | pp. 18-19 |
| **Discussion - Interpretation** | **20** | Give a cautious overall interpretation considering objectives, limitations, multiplicity of analyses, results from similar studies, and other relevant evidence. | pp. 16-19 |
| **Discussion - Generalisability** | **21** | Discuss the generalisability (external validity) of the study results. | pp. 18-19 |
| **Other information - Funding** | **22** | Give the source of funding and the role of the funders for the present study and, if applicable, for the original study on which the present article is based. | No specific funding for this work, as declared in the submission system. |

STROBE = Strengthening the Reporting of Observational Studies in Epidemiology.
